# RNF5 Regulates HERP1 and OS9 Stability and Modulates Processing of an ERAD Substrate

**DOI:** 10.3390/life16101581

**Published:** 2026-09-22

**Authors:** Wenjing Yan, Yanyan Qu, Yongwang Zhong, Christopher A. LeClair, Ganesha Rai, Dingyin Tao, Shengyun Fang

**Affiliations:** 1 Department of Pharmacology and Physiology, University of Maryland School of Medicine, Baltimore, MD 21201, USA; 2 Division of Preclinical Innovation, National Center for Advancing Translational Sciences, National Institutes of Health, Rockville, MD 20850, USA; 3 Program in Oncology, UM Greenebaum Comprehensive Cancer Center, University of Maryland School of Medicine, Baltimore, MD 21201, USA

**Keywords:** endoplasmic reticulum-associated degradation (ERAD), RNF5, HERPUD1 (HERP1), OS9, ubiquitination, unfolded protein response, ER stress, cellular senescence

## Abstract

Endoplasmic reticulum-associated degradation (ERAD) must dynamically adapt to cellular protein-folding demands, yet mechanisms regulating ERAD-associated adaptors remain incompletely defined. Here, we identify the E3 ubiquitin ligase RNF5 as a regulator of the ERAD adaptors HERPUD1 (HERP1) and OS9. RNF5 depletion increased HERP1 and OS9 protein abundance, while semiquantitative endpoint RT-PCR did not reveal obvious changes in their transcript signals. Available cycloheximide-chase experiments were consistent with increased adaptor persistence and more rapid loss of the ERAD-L substrate null Hong Kong α1-antitrypsin (NHK) after RNF5 depletion. In contrast, expression of the catalytically inactive RNF5 C42S mutant was associated with slower NHK loss, indicating that RNF5 depletion and catalytic inactivation produce distinct functional outcomes. Proteomic screening and co-immunoprecipitation supported association of RNF5 with HERP1- and OS9-containing complexes. RNF5 depletion reduced compound 225-induced HERP1 ubiquitination and adaptor loss, while wild-type RNF5 produced stronger HERP1 ubiquitination than C42S, supporting a contribution of RNF5 catalytic activity. RNF5 depletion also attenuated selected tunicamycin-induced UPR markers and 225-associated cytotoxic/apoptosis-related readouts while increasing SA-β-galactosidase- and γH2AX-associated phenotypes. Together, these findings identify RNF5 as a regulator of ERAD-adaptor abundance and NHK processing, while leaving the causal relationship between adaptor regulation and the RNF5-depletion phenotype to be resolved by formal rescue and epistasis studies.

## 1. Introduction

The endoplasmic reticulum (ER) serves as the cellular factory for synthesizing, folding, and modifying proteins destined for the secretory pathway or plasma membrane [1,2]. Maintaining a balanced state of proteostasis—the dynamic equilibrium of protein production and degradation—is vital for cell survival [3]. Accumulation of misfolded or unfolded proteins in the ER, a condition known as ER stress, activates the unfolded protein response (UPR) to restore homeostasis [4,5]. A central effector of this process is ER-associated protein degradation (ERAD), a tightly regulated pathway that retrotranslocates terminally misfolded proteins from the ER lumen to the cytosol for ubiquitination and proteasomal degradation [6]. While the core machinery that recognizes and disposes of misfolded substrates is well characterized, how cells dynamically tune the overall capacity of this pathway to accommodate varying proteostatic loads remains poorly understood [7].

Adaptor proteins such as HERPUD1 (HERP1) and OS9 play pivotal roles in shaping ERAD efficiency. HERP1 functions as a regulated scaffold at the ER membrane, assembling E3 ubiquitin ligases and chaperones into a degradation hub [8,9], while OS9 serves as a lectin that recognizes misfolded glycoproteins and delivers them to the ERAD machinery [10]. Because their abundances likely influence the composition and activity of the ERAD complexes, modulating HERP1 and OS9 levels could provide a rapid, post-transcriptional means of tuning ERAD throughput—potentially providing a post-transcriptional layer of proteostasis regulation beyond the broader transcriptional responses mediated by the UPR [11].

Beyond proteostasis, prolonged or unresolved ER stress can activate pro-apoptotic signaling [4,5] and has also been linked to cellular senescence [12,13]. Chronic ER stress and impaired proteostasis have been implicated as contributors to cellular senescence in aging and degenerative diseases [13,14].

RING-type E3 ubiquitin ligases at the ER membrane contribute critically to ER quality control. RNF5, also known as RMA1, is an ER-associated RING E3 ubiquitin ligase with context-dependent roles in protein quality control. RNF5 promotes the degradation of selected membrane substrates, including misfolded CFTR [15], but can also attenuate ERAD by ubiquitinating the ER-associated scaffold JAMP and limiting its association with p97 and proteasomal components [16]. HERP1 is itself subject to ubiquitin-dependent regulation: POSH-mediated K63-linked ubiquitination regulates HERP1 localization [17], whereas the UBE2G2-gp78 pathway promotes HERP1 degradation during ER-stress recovery [18]. However, whether RNF5 contributes to HERP1 and OS9 turnover under basal conditions or during pharmacologic ERAD perturbation has remained unclear.

In our previous work, we identified compound 225 as an ERAD inhibitor that promotes HERP1 and OS9 degradation and disrupts VDAC-mediated calcium homeostasis at mitochondria-associated membranes [19]. These observations provided a chemical approach for identifying ubiquitin-pathway components involved in adaptor turnover and for investigating how pharmacologic stress alters ERAD-associated machinery.

In this study, we identify RNF5 as a regulator of HERP1 and OS9 protein stability and of the processing of the ERAD-L substrate NHK. RNF5 depletion stabilized both adaptors, enhanced NHK dislocation and clearance, and attenuated selected UPR outputs. Independent HERP1 depletion reduced the NHK-dependent drGFP signal, supporting a functional contribution of HERP1 to NHK processing, although formal RNF5-HERP1 epistasis was not tested. Conversely, the catalytically inactive RNF5 C42S mutant slowed NHK turnover, indicating that RNF5 depletion and catalytic inactivation are distinct perturbations. Compound 225 increased RNF5 association with HERP1-containing complexes, whereas RNF5 depletion reduced 225-induced HERP1 ubiquitination and adaptor loss. Comparison of wild-type RNF5 and C42S further supported a contribution of RNF5 catalytic activity to HERP1 ubiquitination. These findings support an RNF5-dependent layer of regulation of ERAD-associated adaptor abundance and NHK processing while defining the remaining causal questions.

## 2. Materials and Methods

### 2.1. Cell Culture

HeLa (ATCC^®^ CCL-2™) and A-375 (ATCC^®^ CRL-1619) cells were obtained from the American Type Culture Collection (ATCC, Manassas, VA, USA). MCF7ca (MCF7 human breast cancer cells transfected with aromatase gene) cells were kindly provided by Dr. Yun Qiu (University of Maryland, Baltimore, MD, USA). HeLa cells expressing SP-S11-NHK-HA were previously established [20]. All cells were cultured in Dulbecco’s Modified Eagle Medium (DMEM) supplemented with 10% heat-inactivated fetal bovine serum (FBS) and 1% penicillin–streptomycin (100 U/mL penicillin and 100 μg/mL streptomycin). Cells were grown in a humidified incubator at 37 °C with a 5% CO_2_ atmosphere. All cell lines were confirmed to be free of mycoplasma contamination through periodic testing.

### 2.2. Reagents and Antibodies

All antibodies used in this study were obtained from commercial sources, with the following specifications: Anti-CHOP (sc-7351, clone B-3, 1:300), ubiquitin-HRP (sc-8017 HRP, clone P4D1, 1:200) and anti-PARP-1 (sc-8007, 1:500) were obtained from Santa Cruz Biotechnology (Dallas, TX, USA). Anti-AUP1 (A57895, 1:2000) and β-Actin-Peroxidase (A3854, clone AC-15, 1:3000) antibodies were purchased from Millipore Sigma (Burlington, MA, USA). Anti-RNF5 (PA5-71703, 1:1000) antibody was from Invitrogen (Carlsbad, CA, USA). Anti-HERP1 (26739S, 1:1000), anti-OS9 (12497S, 1:1000), anti-UBE2G2 (63182S, clone D8Z4G, 1:500), anti-XBP1s (27901S, clone E8C2Z, 1:1000), anti-p-eIF2α (3398S, clone D9G8, 1:1000), anti-Caspase 3 (#9668, 1:500) and p-Histone H2AX (Ser139) (2577S, 1:300) antibodies were purchased from Cell Signaling Technology (Danvers, MA, USA). Anti-HA-peroxidase (3F10) antibody (12013819001, 1:500) was purchased from Roche (Mannheim, Germany). Anti-BiP (610978, 1:1000), anti-VCP (612182, 1:2000) and anti-calreticulin (612136, 1:100) antibodies were from BD Biosciences (San Jose, CA, USA). Anti-BAG6 (66661-1-1g, 1:2000) antibody was obtained from Proteintech (Rosemont, IL, USA).

### 2.3. Generation of Plasmid Constructs

The pCIneo-HERP1-HA expression vector was generated by cloning the human HERP1 open reading frame (ORF) into the NheI and NotI restriction sites of the pCIneo plasmid. To facilitate this, the HERP1 sequence was amplified using the following primers: Forward 5′-CATTGCTAGCGCCACCATGGAGTCCGAGACCGAAC-3′ and Reverse 5′-GTTGTGCGGCCGCTTATGCGTAATCTGGAACATCATATGGGTAGTTTGCGATGGCTGGG-3′. The construct was verified by DNA sequencing.

### 2.4. siRNA Knockdown

Negative Control siRNA was purchased from Ambion (Austin, TX, USA). Three independent siRNA sequences targeting RNF5 were synthesized by MilliporeSigma, including siRNF5 #1 (5′-AGCUGGGAUCAGCAGAGAG [dT][dT]−3′), siRNF5 #2 (5′-GCGCGACCUUCGAAUGUAA [dT][dT]−3′), and siRNF5 #3 (5′-CGGCAAGAGUGUCCAGUAU [dT][dT]−3′). Transfections were performed using Lipofectamine™ RNAiMAX (Invitrogen) with siRNA diluted in Opti-MEM medium. Following a 72 h incubation, knockdown efficiency was validated via Western blotting. For functional assays, cells were treated with Cycloheximide (CHX) or compound 225 to evaluate downstream effects on protein stability and signaling.

For the HERP1-depletion drGFP experiment, cells were transfected with HERP1-targeting siRNA using the same RNAiMAX-based workflow and were subsequently processed for the NHK-dependent drGFP assay. Knockdown was confirmed by immunoblotting.

### 2.5. Dislocation-Induced Reconstituted GFP (drGFP) Assay [20]

HeLa cells stably expressing SP-S11-NHK-HA were seeded in a 96-well plate at a density of 2 × 10^4^ cells/well. The cells were then transfected with either a negative control siRNA or siRNF5. Following a 3-day incubation, the cells were re-seeded and cultured overnight. Prior to treatment with Bortezomib (BTZ) and/or compound 225, the medium was replaced with fresh culture medium. Real-time protein degradation was monitored via GFP imaging, with images captured every 2 h using an IncuCyte S3 live-cell analysis system.

### 2.6. Cycloheximide (CHX) Chase Assay

Cells were transfected with either a negative control siRNA or siRNF5 for 72 h. To monitor protein stability, cells were then treated with CHX at a final concentration of 50 μg/mL for the indicated time points. At each interval, cell aliquots were harvested and lysed; the resulting lysates were then analyzed via immunoblotting (IB) to determine protein degradation kinetics.

### 2.7. Semiquantitative Endpoint RT-PCR

Total RNA was isolated using TRIzol reagent (Invitrogen) in accordance with the manufacturer’s protocol. The RNA concentration and purity were determined spectrophotometrically. Subsequently, 2 μg of total RNA was reverse-transcribed into cDNA using the ProtoScript^®^ First Strand cDNA Synthesis Kit (New England BioLabs, Ipswich, MA, USA), following the provided instructions for a total reaction volume of 20 μL.

### 2.8. Immunoblotting (IB)

Cells were washed with PBS and lysed for 30 min on ice in a dedicated lysis buffer (10 mM Tris/HCl, 150 mM NaCl, 1 mM EDTA, 1 mM EGTA, 0.2% NP-40 and 0.5% Triton X-100, pH 7.5) supplemented with a protease inhibitor cocktail. The resulting lysates were centrifuged at 20,000× *g* for 10 min at 4 °C. Protein concentrations were determined using the Bradford assay. Equal amounts of protein were separated by 10% SDS-PAGE and transferred onto PVDF membranes. After blocking non-specific binding sites with 5% non-fat milk in TBST for 1 h at room temperature, membranes were incubated with the indicated primary antibodies. Following three washes with TBST, the membranes were incubated with HRP-conjugated secondary antibodies for 1 h at room temperature. Protein bands were visualized using SuperSignal™ West Pico PLUS or SuperSignal™ WestFemto Maximum Sensitivity chemiluminescent substrates (Thermo Fisher Scientific, Waltham, MA, USA).

### 2.9. Immunoprecipitation (IP)

HeLa cells transfected with pCIneo-HERP1-HA were treated with either DMSO (vehicle control) or compound 225 for 2 h. Following treatment, cells were harvested and lysed in a specialized IP lysis buffer (150 mM NaCl, 10 mM Tris/HCl, pH 7.4, 1 mM EDTA, 1 mM EGTA, 0.2% NP-40, and a protease inhibitor cocktail). For each IP reaction, 600 µg of total protein was adjusted to a final volume of 700 µL using lysis buffer. The lysates were then incubated with anti-HA affinity beads for 2 h at 4 °C under constant rotation. Following incubation, the beads were collected, the supernatant was discarded, and the resin was washed three times with 700 µL of lysis buffer. The resulting immunoprecipitates were eluted and processed for immunoblotting (IB).

For reciprocal co-immunoprecipitation, cells expressing HA-RNF5 were treated with vehicle or compound 225 as indicated and subjected to anti-HA immunoprecipitation under the same lysis and wash conditions, recovered HERP1 and OS9 were assessed by immunoblotting.

### 2.10. Analysis of Protein Ubiquitination in Cells by Re-Immunoprecipitation (reIP)

HERP1 ubiquitination was assessed by reIP under denaturing condition. Cells stably expressing HA-HERP1 were treated with 2 μM BTZ with or without 225 for 3 h. Following treatment, cells were lysed under native conditions and subjected to an initial round of immunoprecipitation (IP) using anti-HA conjugated beads for 2 h at 4 °C. To eliminate non-covalently associated proteins and ensure the detection of direct HERP1 ubiquitination, the primary immunoprecipitates were eluted and denatured in 2% SDS by boiling. The beads were subsequently removed by centrifugation. The resulting supernatants were diluted 20-fold in native lysis buffer (to reduce the SDS concentration) and subjected to a second round of IP (reIP) with anti-HA beads. The final denatured precipitates were then analyzed by immunoblotting (IB) using anti-ubiquitin antibodies.

For the wild-type and C42S comparison, cells expressing HA-HERP1 together with vector, wild-type RNF5, or RNF5 C42S were processed with the same denaturing re-immunoprecipitation workflow, and HERP1 ubiquitination was assessed by anti-ubiquitin immunoblotting.

### 2.11. Mass Spectrometry Proteomics [21]

For proteomic analysis, HeLa cells transiently expressing pCIneo-HERP1-HA were treated with or without 225, followed by anti-HA immunoprecipitation. The resulting IP samples were processed using an on-beads digestion protocol. Briefly, beads were washed five times with 25 mM ammonium bicarbonate and then reduced with 15 mM Tris (2-carboxyethyl) phosphine (TCEP) at 55 °C for 30 min. After cooling, proteins were alkylated with 15 mM iodoacetamide (IAA) in the dark for 30 min. Protein digestion was performed by adding 50 ng of trypsin and incubating at 37 °C overnight. The reaction was quenched with 1% formic acid (FA), and the supernatant was collected. To maximize peptide recovery, beads were further incubated with 60% acetonitrile (ACN) containing 0.1% FA for 5 min, and the second supernatant was pooled with the first. Samples were dried via SpeedVac and resuspended in 15 µL of sample buffer (97.9% water, 2% ACN, and 0.1% FA). LC-MS/MS analysis was performed using an UltiMate 3000-nano LC system coupled to an Orbitrap Fusion Lumos Tribrid mass spectrometer (Thermo Fisher Scientific). Peptides were loaded onto a C18 trap column (Acclaim PepMap 100 C18, 75 µm × 2 cm) and separated on a 30 cm analytical column (CoAnn Technologies, Richland, WA, USA) using a 120 min method (~97 min gradient) starting at 98% buffer A (0.1% FA in MS-grade water) at a flow rate of 250 nL/min. The mobile phase was maintained at 2% buffer B (80% ACN, 19.9% water, 0.1% FA) for 8 min, 2–10% B for 2 min, 10–32% B for 84 min, 32–90% B for 1 min and 90% B for 10 min, followed by re-equilibration of the column with 2% B for 15 min. The mass spectrometer operated in data-dependent acquisition (DDA) mode. Full MS scans (*m*/*z* 350–2000) were acquired in the Orbitrap at 60,000 resolution, followed by HCD fragmentation (28% NCE) and MS2 detection in the IonTrap. Raw data were processed using Proteome Discoverer (v2.4) with the Sequest HT search algorithm against the Swiss-Prot human database. Search parameters included a precursor mass tolerance of 10 ppm, a fragment mass tolerance of 0.5 Da, and a maximum of two missed cleavage sites. Carbamidomethylation of cysteine was set as a fixed modification, while methionine oxidation and N-terminal acetylation were set as variable modifications. Label-free quantification (LFQ) was performed using the Precursor Ions Quantifier node, and protein abundances were normalized by total peptide amount with a false discovery rate (FDR) threshold of <1%. The HERP1 AP-MS comparison was performed as a single biological experiment (one sample per condition) and was used only as an exploratory screen. Accordingly, no inferential statistical analysis or quantitative enrichment claim was made from this dataset.

### 2.12. Cell Growth/Confluence Assay

Cells were transfected with control or RNF5-targeting siRNA for 72 h and subsequently seeded into 96-well plates at 3 × 10^3^ cells/well. After overnight attachment, cells were treated with vehicle or the indicated concentrations of compound 225. Cell growth was monitored using an IncuCyte S3 live-cell analysis system (Sartorius, Göttingen, Germany), with phase-contrast images acquired every 4 h. Cell growth was quantified as percent phase confluence using the IncuCyte image-analysis software (2022A).

### 2.13. Cell Viability Assay

Cell viability was assessed using a WST-based assay. Cells transfected with control or RNF5-targeting siRNAs were seeded into 96-well plates at 3 × 10^3^ cells/well and treated with vehicle or compound 225 as indicated. After treatment, 10 μL of WST reagent was added to each well and incubated for 1 h at 37 °C. Absorbance was measured at 450 nm using a microplate reader. Viability was normalized to the corresponding vehicle-treated control.

### 2.14. Senescence-Associated β-Galactosidase Assay

Senescence-associated β-galactosidase activity was assessed using the CellEvent™ Senescence Green Detection Kit (Invitrogen, Carlsbad, CA, USA). Cells were seeded in 96-well plates and subjected to the indicated siRNA treatments. Cells were fixed with 2% paraformaldehyde for 10 min at room temperature and washed twice with PBS. The CellEvent Senescence Green reagent was prepared according to the experimental protocol and applied to the cells. Cells were incubated at 37 °C for 2 h in the absence of CO_2_ and subsequently imaged by IncuCyte. SA-β-galactosidase-positive cells were quantified from the acquired fluorescence images and expressed as the percentage of positive cells.

### 2.15. Statistical Analysis

Data are presented as mean ± SEM unless otherwise indicated. Normality was assessed using the Shapiro–Wilk test applied to model residuals or paired differences, as appropriate. Normally distributed two-group datasets were analyzed using two-tailed Student’s *t*-tests, whereas datasets that did not satisfy the normality assumption were analyzed using an appropriate non-parametric test or transformed before analysis, as specified in the corresponding figure legend.

Time-course experiments were analyzed using repeated-measures ANOVA with the relevant interaction term, with Greenhouse–Geisser correction applied where appropriate. Experiments involving crossed independent factors, including siRNA × drug-treatment designs, were analyzed using two-way ANOVA with the interaction term explicitly evaluated. For the IncuCyte growth experiment, the complete growth trajectories were additionally summarized by area-under-the-curve analysis before factorial statistical testing. Multiple-comparison correction was applied where appropriate. The statistical test and sample size used for each quantitative experiment are specified in the corresponding figure legend or Source Data.

## 3. Results

### 3.1. RNF5 Depletion Increases the ERAD-Associated Adaptor Proteins HERP1 and OS9

To investigate whether RNF5 regulates the abundance of ERAD-associated adaptor proteins, we examined HERP1 and OS9 following siRNA-mediated RNF5 depletion. Three independent RNF5-targeting siRNAs increased HERP1 and OS9 protein abundance in HeLa cells (Figure 1A). Semiquantitative endpoint RT-PCR did not reveal an obvious difference in HERPUD1 or OS9 transcript signal following RNF5 depletion (Figure 1B). Because this assay was not quantitative, we do not use it to exclude more modest transcriptional effects. In the available CHX-chase experiments, HERP1 and OS9 signals declined more slowly following RNF5 depletion, consistent with increased protein persistence under these conditions (Figure 1C,D).

### 3.2. RNF5 Depletion and Catalytic Inactivation Differentially Regulate NHK Processing

We next examined the processing of the ERAD-L model substrate NHK. RNF5 knockdown with three independent siRNAs accelerated NHK loss in CHX-chase assays (Figure 2A–C). Consistently, RNF5 depletion increased NHK dislocation as measured by the NHK-dependent drGFP assay under bortezomib-treated conditions (Figure 2D,E). Because these experiments use an NHK-derived ERAD-L reporter, the results establish an effect on this substrate rather than on all ERAD branches. To examine whether HERP1 itself is functionally relevant to NHK processing, we independently depleted HERP1 and observed a reduction in the NHK-dependent drGFP signal (Figure 2F,G). This finding supports a functional contribution of HERP1 to NHK processing but does not constitute formal RNF5-HERP1 epistasis. We also compared RNF5 depletion with catalytic inactivation. In contrast to RNF5 depletion, expression of RNF5 C42S was associated with slower NHK loss than vector control (Figure 2H). Thus, depletion of RNF5 and expression of an inactive RNF5 mutant produce distinct effects on NHK processing.

### 3.3. Compound 225 Increases RNF5 Association with HERP-Containing Complexes

Our previous work identified the small molecule 225 as an ERAD inhibitor that promotes loss of the adaptor proteins HERP1 and OS9 [19]. To identify ubiquitin-pathway components associated with this response, we performed HERP1-HA immunoprecipitation followed by label-free mass spectrometry. This analysis was used as a discovery screen. RNF5 was identified as a candidate in the 225-treated HERP1-HA immunoprecipitate and was selected for follow-up by co-immunoprecipitation (Figure 3A). Co-immunoprecipitation confirmed increased recovery of endogenous RNF5 with HERP1-HA after 225 treatment (Figure 3B). Reciprocal immunoprecipitation of HA-RNF5 also recovered HERP1 and OS9 (Figure 3C), providing additional biochemical support for the association of RNF5 with HERP1 or OS9-containing complexes. These experiments establish complex association but do not by themselves demonstrate direct protein–protein binding.

### 3.4. RNF5 Contributes to 225-Induced HERP1 Ubiquitination and Adaptor Degradation

To determine whether RNF5 contributes to 225-induced HERP1 ubiquitination and adaptor loss, we first monitored HERP1 and OS9 turnover using CHX-chase assays. Compound 225 was associated with more rapid loss of both adaptor signals during CHX treatment (Figure 4A,B). RNF5 depletion prevented the 225-induced reduction in steady-state HERP1 and OS9 abundance in both HeLa and MCF7ca cells (Figure 4C,D). In denaturing re-immunoprecipitation assays, 225 increased HERP1 polyubiquitination, whereas the ubiquitination signal was strongly reduced following RNF5 depletion (Figure 4E), supporting an RNF5-dependent contribution to HERP1 ubiquitination. To further examine catalytic dependence, we compared wild-type RNF5 with the catalytically inactive C42S mutant in the denaturing HERP1 re-immunoprecipitation assay. Wild-type RNF5 produced a stronger HERP1 ubiquitination signal than C42S (Figure 4F), supporting a contribution of RNF5 catalytic activity to HERP1 ubiquitination. These data do not establish whether OS9 is directly ubiquitinated by RNF5.

### 3.5. RNF5 Depletion Attenuates Selected UPR Markers Under Tunicamycin-Induced ER Stress

Given that enhanced processing of ERAD substrates could reduce the accumulation of misfolded proteins, we examined selected UPR markers under tunicamycin (Tm)-induced ER stress. RNF5-depleted cells showed reduced PERK activation-associated mobility shift, XBP1s protein accumulation, and CHOP expression (Figure 5A,B). These findings indicate that RNF5 depletion is associated with attenuation of selected PERK- and IRE1-associated UPR outputs under tunicamycin-induced stress.

### 3.6. RNF5 Depletion Attenuates 225-Associated Cytotoxic Responses but Increases SA-β-Galactosidase- and γH2AX-Associated Phenotypes

Finally, we examined cellular phenotypes associated with RNF5 depletion and compound 225. Live-cell imaging showed that RNF5 knockdown attenuated 225-induced suppression of cell growth (Figure 6A), and endpoint viability measurements showed reduced 225-associated loss of viability following RNF5 depletion (Figure 6C). Immunoblotting showed reduced PERK-eIF2α signaling and reduced cleavage-associated changes in PARP, Caspase-3, and Caspase-8 after RNF5 depletion (Figure 6B). These immunoblot readouts are described as apoptosis-associated markers rather than as an independent quantitative apoptosis assay. In separate experiments, RNF5 depletion increased the proportion of SA-β-galactosidase-positive cells in A375 and HeLa cells (Figure 6E,F) and increased γH2AX phosphorylation (Figure 6G).

## 4. Discussion

Cellular proteostasis depends on coordinated protein synthesis, folding, and degradation [22]. Although the core ERAD machinery is well characterized, how cells regulate the abundance and organization of ERAD-associated components remains incompletely understood [23]. Here, we identify RNF5 as a regulator of HERP1 and OS9 abundance that influences processing of the tested NHK ERAD-L reporter and selected cellular stress responses.

RNF5 depletion increased HERP1 and OS9 protein abundance, while semiquantitative endpoint RT-PCR did not reveal obvious changes in their transcript signals. The available CHX-chase experiments were consistent with increased persistence of both adaptors following RNF5 depletion. RNF5 depletion also increased NHK-dependent drGFP dislocation, whereas independent HERP1 depletion reduced the drGFP signal, supporting a functional contribution of HERP1 to NHK processing. However, because combined RNF5/HERP1 or RNF5/OS9 depletion was not performed, adaptor regulation and altered NHK processing cannot yet be placed in a linear causal pathway.

These findings are consistent with the context-dependent functions of RNF5 in ER quality control. RNF5 promotes degradation of selected membrane substrates such as CFTR [15], but RNF5-dependent ubiquitination of JAMP can also restrain ERAD activity [16]. HERP1 is regulated by additional ubiquitin pathways, including POSH-mediated K63-linked ubiquitination [17] and UBE2G2-gp78-mediated polyubiquitination during ER-stress recovery [18]. In the present study, 225-induced HERP1 ubiquitination was reduced by RNF5 depletion, and wild-type RNF5 produced stronger HERP1 ubiquitination than the catalytically inactive C42S mutant, supporting a contribution of RNF5 catalytic activity to HERP1 ubiquitination. Direct OS9 ubiquitination was not examined, and the RNF5-dependent change in OS9 may therefore be direct or secondary to changes in HERP1-containing complexes.

In our previous study, Ruan et al. (2022) [24], we used the same HeLa NHK-drGFP reporter and reported an increased drGFP signal following RNF5 knockdown under bortezomib-treated conditions, as well as inhibition of NHK degradation by an E3-inactive RNF5 RING mutant. Thus, the increased drGFP signal observed here is consistent with our previous report rather than unique to the present study. In the current study, the CHX-chase experiment with RNF5 C42S was consistent with slower NHK loss, whereas RNF5 depletion was associated with more rapid NHK loss. Together, these observations indicate that RNF5 depletion and catalytic inactivation are not functionally equivalent perturbations. A possible explanation is that an inactive RNF5 protein retains interaction or scaffolding functions and exerts a dominant-negative effect, whereas siRNA-mediated depletion removes the RNF5 protein. This remains a hypothesis that will require formal siRNA-resistant rescue experiments to resolve.

The present study also extends our previous characterization of compound 225 as a VDAC-binding ERAD inhibitor that disrupts calcium homeostasis [19]. Compound 225 increased RNF5 recovery in HERP1-containing complexes, whereas RNF5 depletion reduced 225-induced HERP1 ubiquitination and prevented the loss of HERP1 and OS9 abundance. These findings identify RNF5 as a component of the cellular response to 225, but not as a demonstrated direct molecular target. Likewise, although 225 perturbs VDAC-dependent calcium signaling, the present data do not establish that altered Ca^2+^ flux directly recruits RNF5; this relationship remains correlative.

RNF5 depletion was also associated with attenuation of selected UPR outputs and 225-associated apoptosis-related readouts. More broadly, ER proteostasis and UPR dysregulation have been implicated in cancer, neurodegenerative disorders, and other diseases [11,25,26], and RNF5 has been linked to cellular responses to ER-stress-inducing chemotherapy [27]. However, the present experiments do not establish disease-specific consequences of RNF5-dependent HERP1 and OS9 regulation.

Unexpectedly, RNF5 depletion increased SA-β-galactosidase activity and γH2AX phosphorylation. These findings suggest that RNF5 depletion can have divergent effects on different stress-associated phenotypes. Because these markers alone do not establish irreversible senescence, DNA damage, or a cell-fate switch, we restrict the interpretation to the measured SA-β-galactosidase- and γH2AX-associated phenotypes [28,29].

Several limitations define the scope of the study. The functional substrate assays are restricted to NHK-derived ERAD-L reporters and should not be generalized to ERAD-M or ERAD-C substrates. Formal RNF5 rescue and RNF5/HERP1 or RNF5/OS9 epistasis experiments were not performed, and OS9 ubiquitination was not directly assessed. Future studies will be required to define how RNF5 activity is regulated and how it integrates with other ubiquitin-pathway components and stress-response pathways [30].

In summary, our findings identify RNF5 as a regulator of HERP1 and OS9 abundance that modulates processing of the tested NHK ERAD-L substrate. The distinct effects of RNF5 depletion and catalytic inactivation, together with the HERP1 ubiquitination data, support a role for RNF5 in adaptor regulation while leaving formal rescue and epistasis as important tests of causality. A working model summarizing these findings is shown in Figure 7.

## Figures and Tables

**Figure 1:**
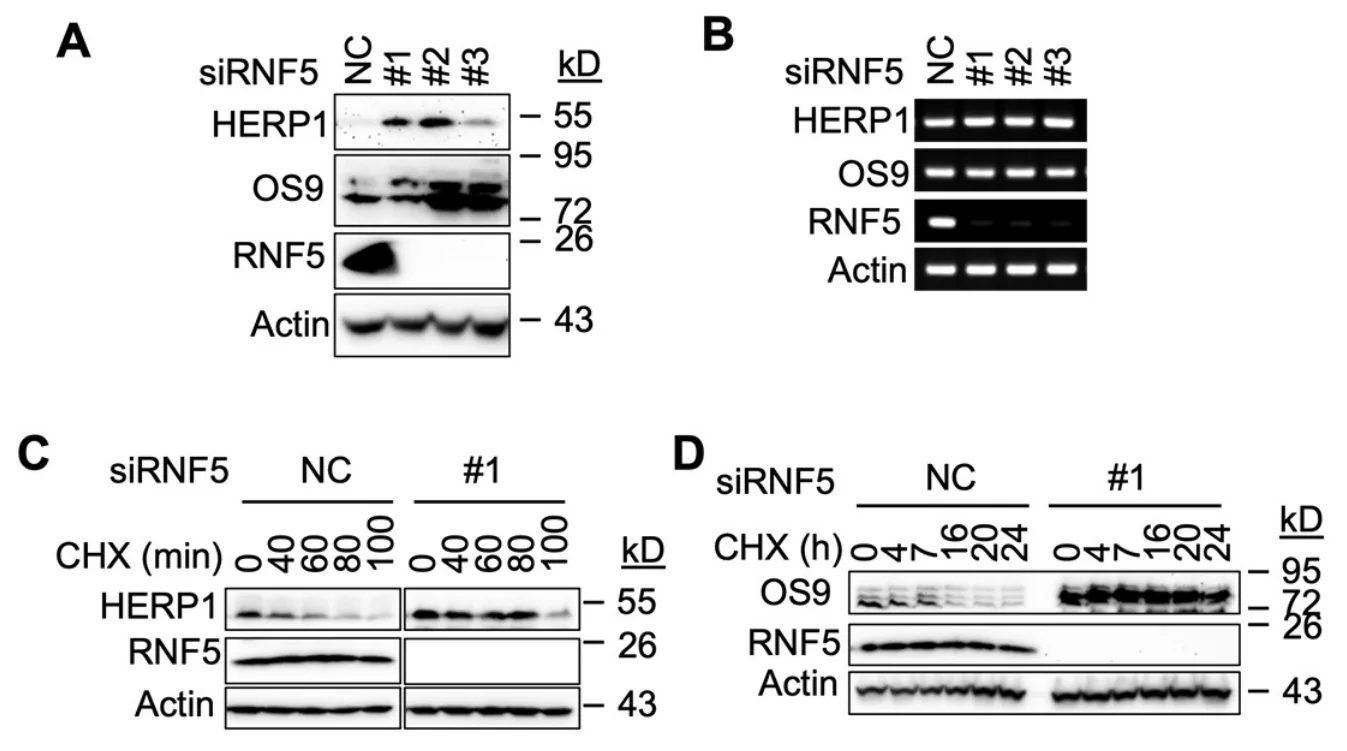
RNF5 depletion increases the ERAD-associated adaptor proteins HERP1 and OS9. (**A**) Immunoblot analysis of endogenous HERP1 and OS9 in HeLa cells transfected with control siRNA or three independent RNF5-targeting siRNA (#1, #2 and #3). (**B**) Semiquantitative endpoint RT-PCR of HERPUD1, OS9, and RNF5 transcript signals following RNF5 depletion. The assay did not reveal an obvious difference in HERPUD1 or OS9 signal under the conditions tested and is not interpreted as a quantitative measurement of transcript abundance. (**C**,**D**) CHX-chase assays showing slower turnover of HERP1 (**C**) and OS9 (**D**) following RNF5 depletion.

**Figure 2:**
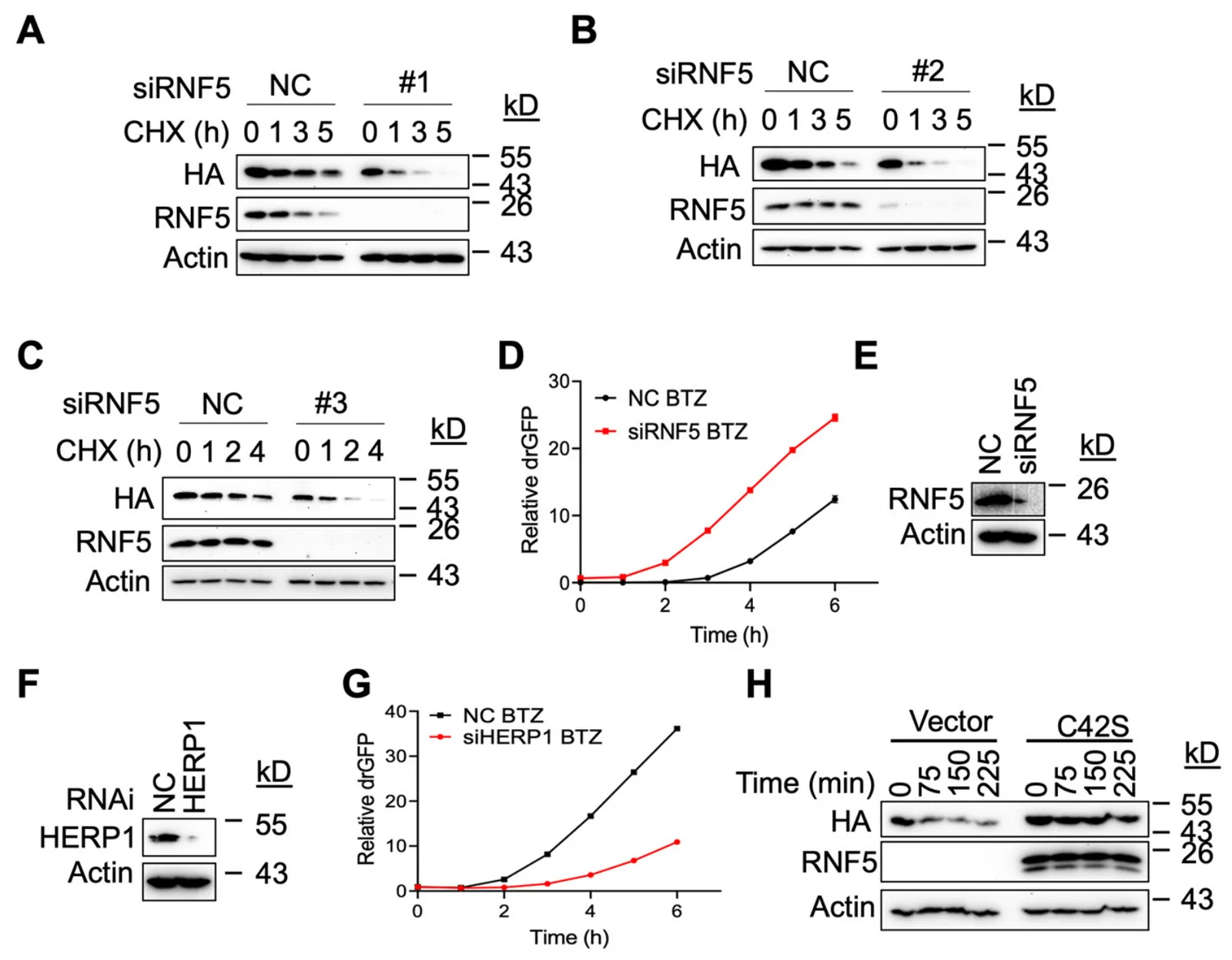
RNF5 depletion and catalytic inactivation differentially regulate NHK processing. (**A**–**C**) CHX-chase assays monitoring NHK-HA turnover after depletion of RNF5 with three independent siRNAs (#1–#3). (**D**) Time-course analysis of NHK-drGFP reporter signal in control and RNF5-depleted cells. Data are presented as mean ± SEM from four replicates. Statistical analysis was performed using two-way repeated-measures ANOVA with Greenhouse–Geisser correction. A significant time × siRNA interaction was observed F (1.083, 6.499) = 230.41, *p* = 2.26 × 10^−6^. Normality of model residuals was assessed using the Shapiro–Wilk test. (**E**) Immunoblot confirmation of RNF5 depletion for the drGFP experiment. (**F**) Immunoblot confirmation of HERP1 depletion. (**G**) NHK-dependent drGFP signal after HERP1 depletion under the indicated bortezomib and compound 225 conditions. HERP1 depletion reduced the drGFP signal, supporting a functional contribution of HERP1 to NHK processing. (**H**) NHK-HA CHX time course in cells expressing vector or the catalytically inactive RNF5 C42S mutant. C42S expression slowed NHK loss relative to vector control. The C42S experiment is interpreted as evidence that catalytic inactivation and RNF5 depletion are phenotypically distinct rather than as a formal rescue experiment.

**Figure 3:**
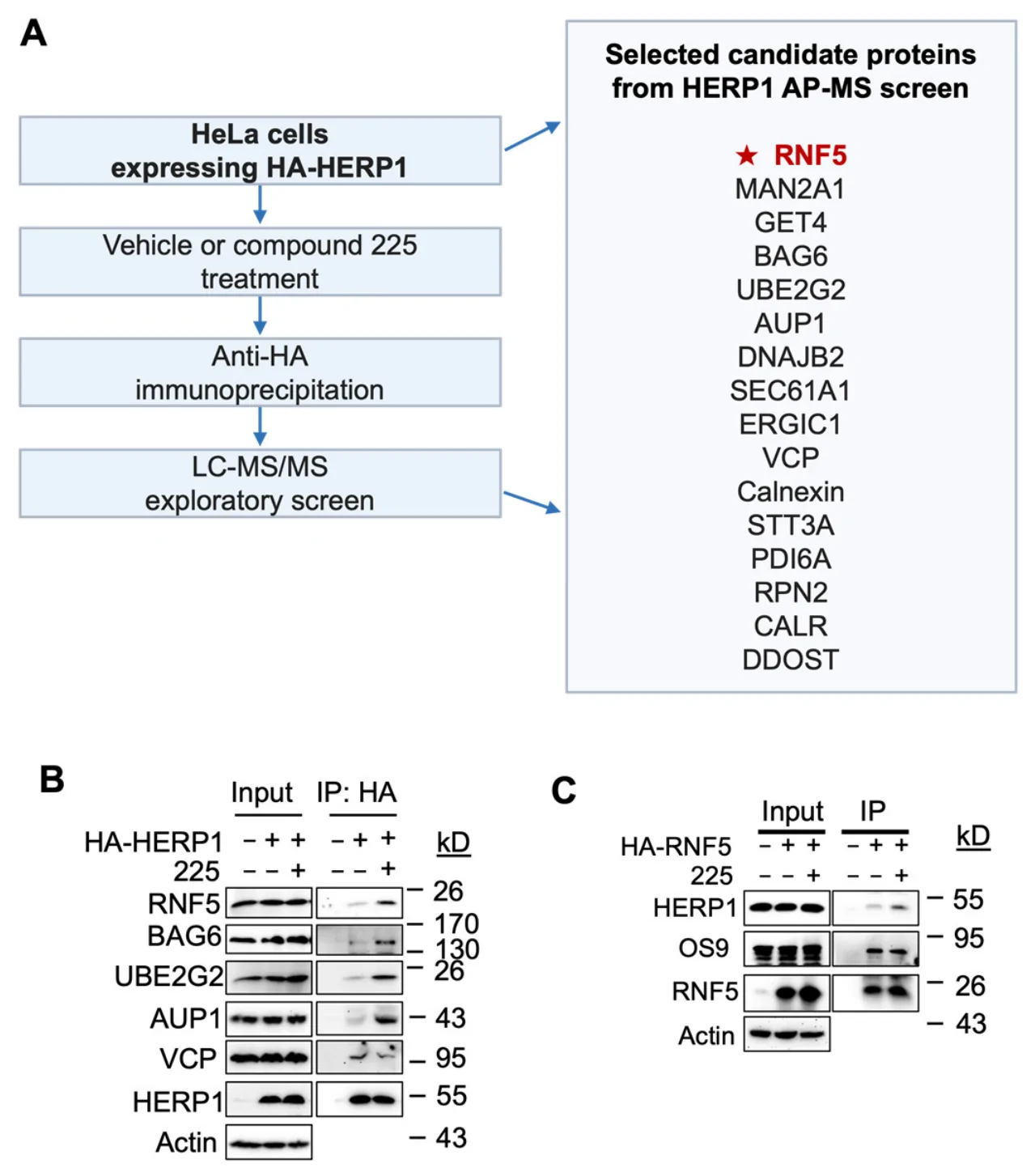
Compound 225 increases RNF5 association with HERP1-containing complexes. (**A**) Exploratory label-free AP-MS screen of HERP1-HA immunoprecipitates from vehicle- and 225-treated cells. The dataset is presented as hypothesis-generating rather than as a formal quantitative enrichment analysis. Arrows indicate the experimental workflow, and the red star highlights RNF5 as the candidate selected for follow-up analysis. (**B**) Co-immunoprecipitation showing increased recovery of endogenous RNF5 with HERP1-HA after 225 treatment. (**C**) Reciprocal HA-RNF5 immunoprecipitation showing recovery of HERP1 and OS9 in RNF5-containing complexes under the indicated conditions. Input and immunoprecipitated fractions were analyzed by immunoblotting.

**Figure 4:**
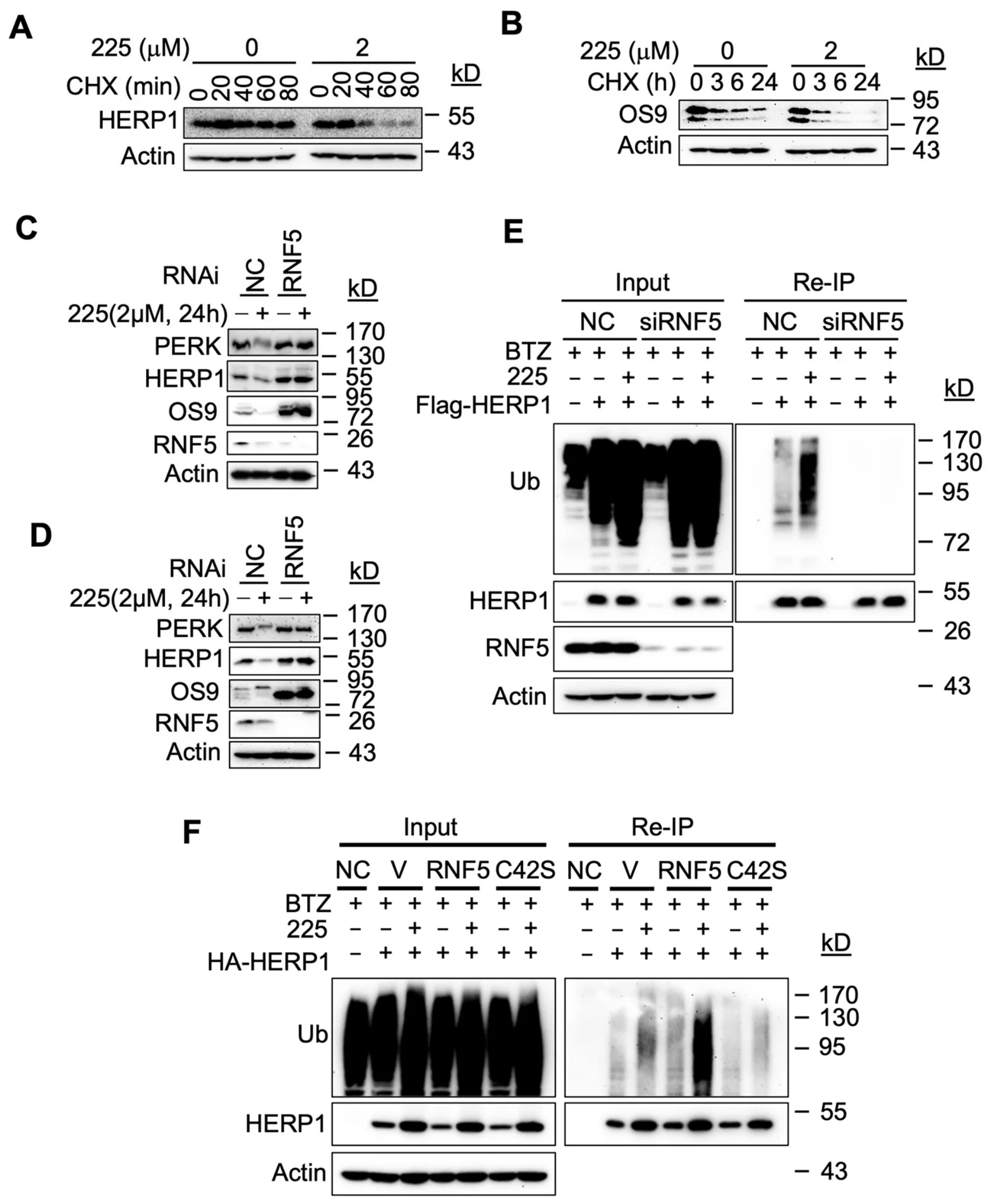
RNF5 contributes to 225-induced HERP1 ubiquitination and adaptor degradation. (**A**,**B**) Compound 225 was associated with more rapid loss of HERP1 (**A**) and OS9 (**B**) signals during CHX treatment. (**C**,**D**) RNF5 depletion prevents the 225-induced reduction in HERP1 and OS9 abundance in HeLa (**C**) and MCF7ca (**D**) cells. (**E**) Denaturing re-immunoprecipitation assay showing increased HERP1 ubiquitination after 225 treatment and reduced ubiquitination following RNF5 depletion. (**F**) Denaturing HERP1 re-immunoprecipitation comparing vector, wild-type RNF5, and the catalytically inactive RNF5 C42S mutant. Wild-type RNF5 produced stronger HERP1 ubiquitination than C42S under the tested conditions. These data support a contribution of RNF5 catalytic activity to HERP1 ubiquitination but are not a substitute for a formal siRNA-resistant rescue experiment.

**Figure 5:**
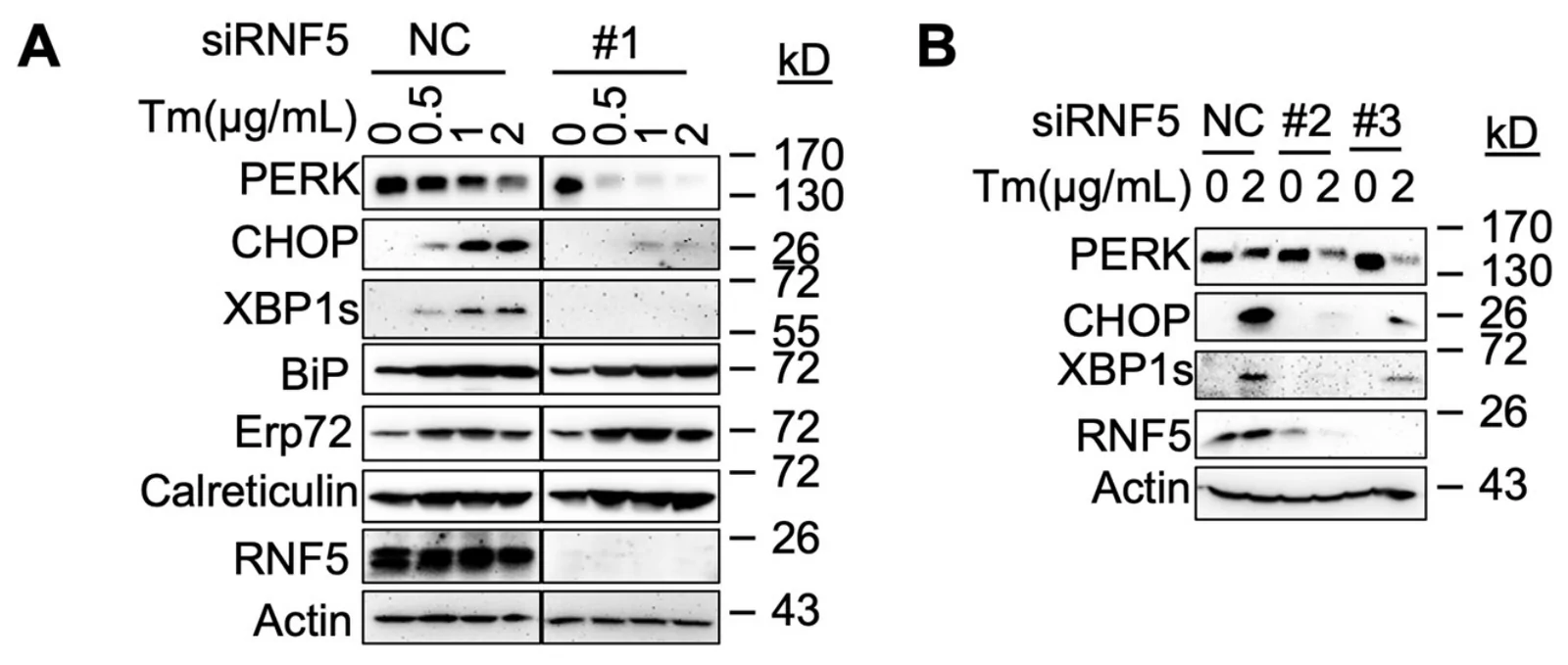
RNF5 depletion attenuates selected UPR markers under tunicamycin-induced ER stress. (**A**,**B**) Immunoblot analysis of UPR signaling markers in control and RNF5-depleted cells treated with tunicamycin (Tm) for the indicated doses and siRNAs. Representative blots are shown from at least three independent experiments. #1, #2, and #3 indicate three independent siRNAs targeting RNF5.

**Figure 6:**
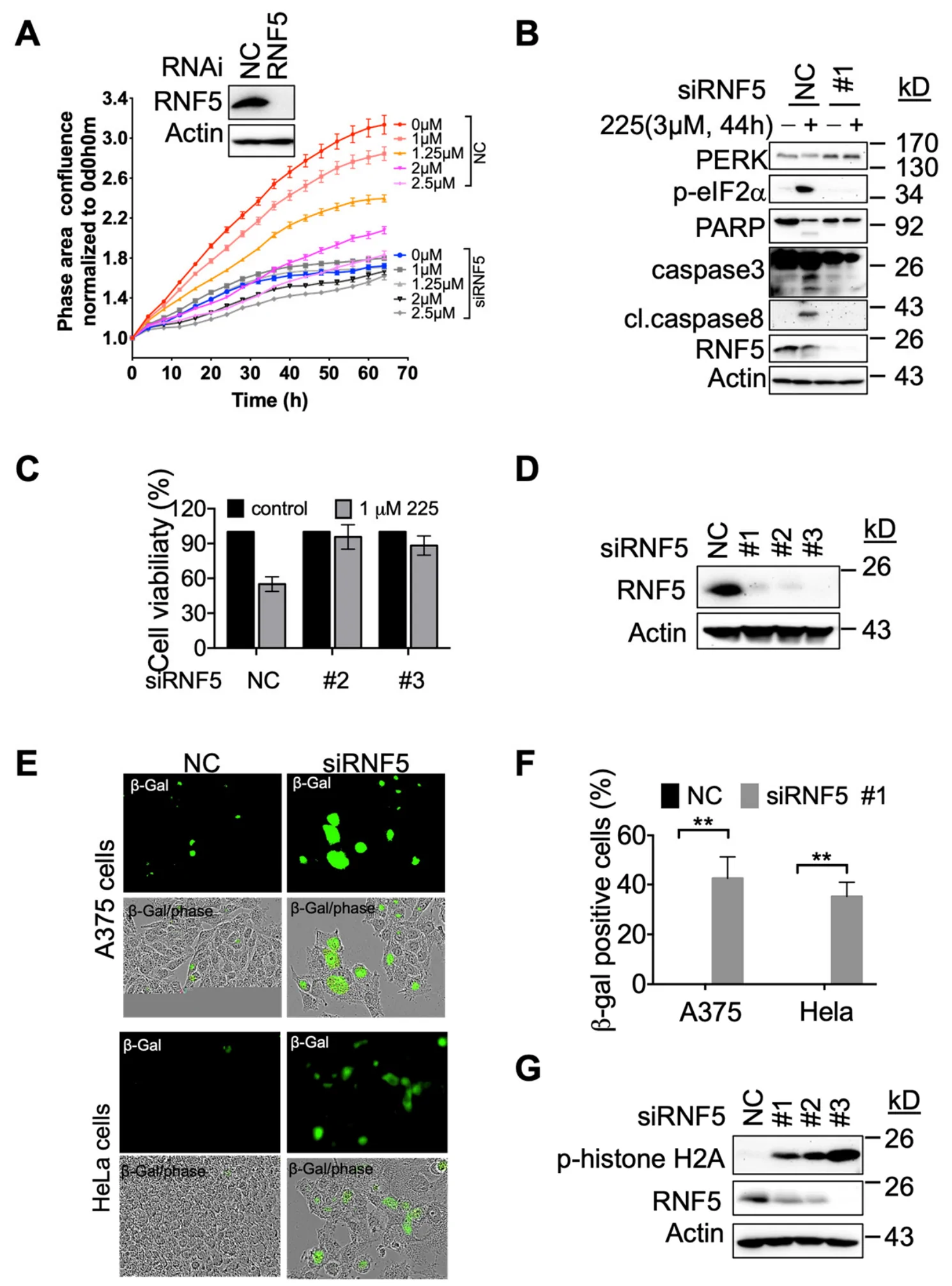
RNF5 depletion attenuates 225-associated cytotoxic responses but increases SA-β-galactosidase- and γH2AX-associated phenotypes. (**A**) Real-time cell growth analysis of control and RNF5-depleted HeLa cells treated with the indicated concentrations of compound 225 using IncuCyte live-cell imaging. Data are presented as mean ± SEM from three independent replicates. The complete 0–64 h growth trajectories were summarized by area-under-the-curve analysis and evaluated by two-way ANOVA with RNF5 siRNA and compound concentration as factors. A significant siRNA × drug interaction was detected (*p* = 1.90 × 10^−4^). Normality of model residuals was assessed using the Shapiro–Wilk test. (**B**) Immunoblot analysis of PERK-eIF2α signaling and apoptosis-associated markers, including PARP, Caspase-3, and cleaved Caspase-8, following 225 treatment. (**C**) Endpoint viability measurements following 225 treatment in cells transfected with the indicated RNF5-targeting siRNAs. (**D**) Immunoblot confirmation of RNF5 depletion. (**E**,**F**) Representative images and quantification of SA-β-galactosidase-positive cells following RNF5 depletion (siRNF5 #1) in A375 and HeLa cells. *n* = 3 biological replicates. Statistical significance was determined using an unpaired two-tailed Student’s *t*-test, ** *p* < 0.01. (**G**) Immunoblot analysis of phosphorylated H2AX following RNF5 depletion. The SA-β-galactosidase and γH2AX measurements are interpreted as marker-level phenotypes and not as sufficient evidence by themselves for irreversible senescence, DNA damage, or cell-fate switching. #1, #2, and #3 indicate three independent siRNAs targeting RNF5.

**Figure 7:**
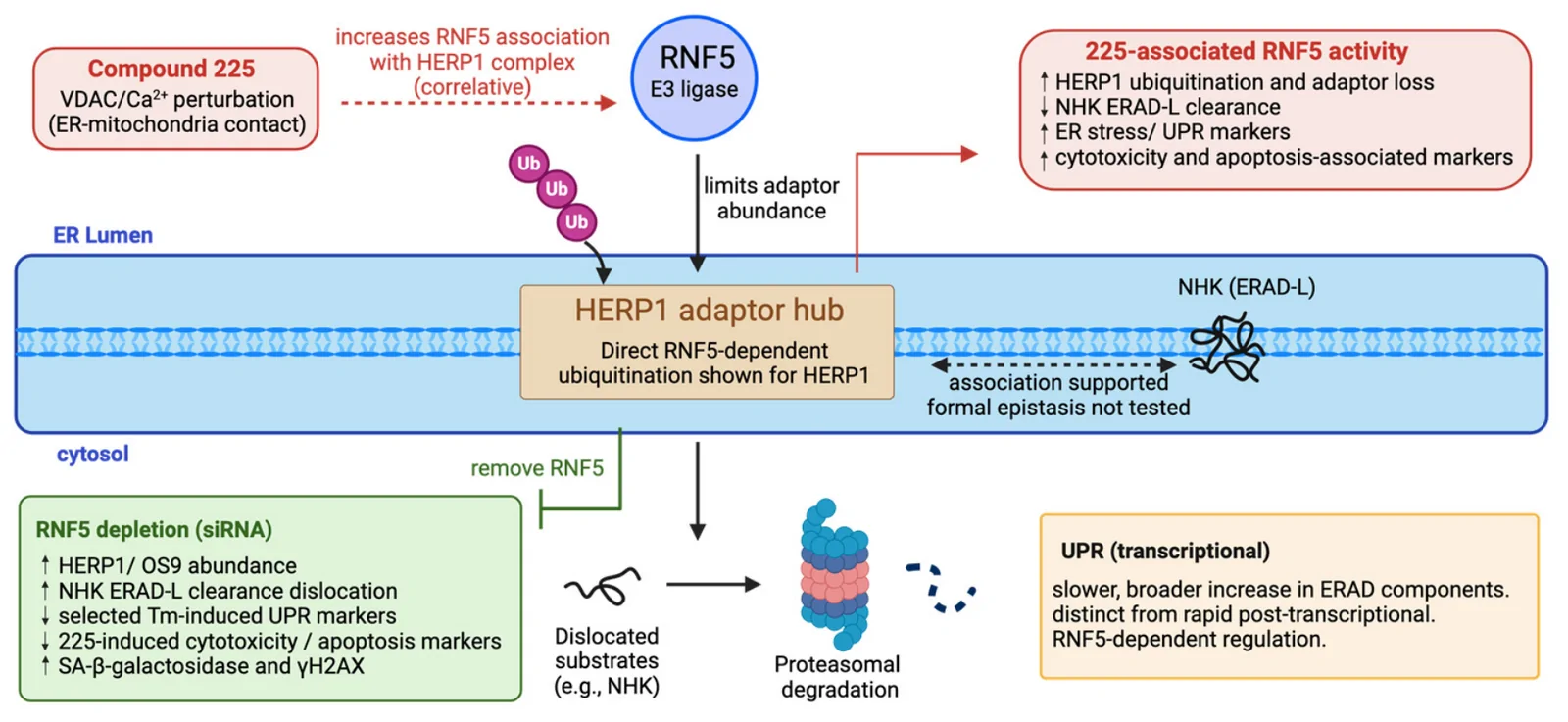
Working model of RNF5-dependent regulation of ERAD-associated adaptor abundance and NHK processing. Dashed relationships indicate correlative or not-yet-mechanistically established steps. Created in BioRender. Yan, W. (2026), https://BioRender.com/qhai8fq.

## Data Availability

The mass spectrometry proteomics data, including raw and processed files, have been deposited to the ProteomeXchange Consortium via the PRIDE partner repository with the dataset identifier PXD081807 [31].

## Abbreviations

The following abbreviations are used in this manuscript:

ERAD	Endoplasmic reticulum-associated degradation
BTZ	Bortezomib
CHX	Cycloheximide
UPR	Unfolded protein response
